# Teeth and the Worker

**Published:** 1920-08

**Authors:** James Burnet

**Affiliations:** Edinburgh, Scotland


### TEETH AND THE WORKER

JAMES BURNET, M.A., M.D., EDINBURGH, SCOTLAND

The establishment of industrial dental clinics in connection with the larger industries of Scotland is an important problem now confronting the dental profession, and requiring early attention. The wise and humanitarian solution of this problem will without doubt have historic effect upon the future attitude of government and official bodies toward the profession of dentistry. Whether that attitude will be antagonistic, or one of sympathy and friendship, will depend upon dentistry itself—upon whether the profession recognizes its full public duty or concerns itself primarily with what it may conceive to be its own affairs. Dr. Burnet has given the whole problem of industrial dentistry a great deal of thought, and this paper, which recently appeared in the *Journal of Industrial Hygiene*, will doubtless be read with much interest.—(Editor, *Oral Health*, from which this article is reprinted.)

The importance of good teeth can not be overestimated. In this connection prophylaxis is vastly superior to any curative measures which may be adopted once the teeth begin to show signs of decay. Carious teeth are always a source of ill health. Such conditions as alveolar abscess, enlarged cervical glands which may eventually become tuberculosis, inflammatory affections of the throat, eye troubles, pernicious anaemia, arthritic disease, and conditions due to septic absorption may all be caused by the presence of unhealthy teeth. In fact, there is no limit to the conditions which may arise to threaten the health of the individual when the teeth begin to show signs of decay. Bad teeth eventually lead to imperfect mastication, and this, in turn, causes indigestion which produces malnutrition with all its attendant consequences, such as anaemia, general feebleness, lack of energy, drowsiness by day and sleeplessness at night, headaches, depression of spirits, and so on. Such conditions do not make for strength on the part of the worker whose health becomes more and more impaired as a result. It is true that in certain cases the individual has a store of reserve energy on which he can fall back, but this is by no means always the case. In the average worker bad teeth mean impaired health and loss of work or diminished output. It is, therefore, of the utmost importance that in all welfare work we must not lose sight of the value of healthy teeth to the worker, whatever his or her age may be.

Out of 10,000 boys and girls between the ages of 14 and 16 years, whom I examined in factories and workshops for the home office during the past few years, I have found a very small percentage with perfect teeth. Even where the teeth are obviously carious, no attempt, in many instances, has been made to deal with them during school age. Some of those who had very good teeth admitted that they either never brushed them at all or did so at very irregular intervals. In fact, I am inclined to think that many dentifrices now sold to the public may be actually injurious to the teeth if applied too frequently. In some cases the teeth were in a very bad condition indeed, and the health of the young person was obviously impaired.

The working classes are constantly drugging their children with such remedies as grey powder, chemical food and other mixtures containing iron, as well as patent cough mixtures, all of which tend in time to destroy the teeth. This custom is fostered by free hospital advice and medicine. Very often childern attend a hospital or dispensary long after it is necessary, and continue swallowing tonic mixtures and "cough bottles" which can only do harm.

During the war it was a notorious fact that the largest consumption of confections was by the working classes. Women and children greedily ate up all they could get, irrespective of the cost. Now that the war is over the same thing goes on. The result is that the teeth become markedly affected. Sugar is good as a fat producer, but it is extremely bad for the teeth.

Tobacco smoking was prevalent amongst school-children during the war, but it has become much more common since. It is quite common to see boys of 10 years smoking cigarettes, while in factories it is quite unusual to find a boy who does not smoke. Unfortunately, the kind of cigarettes smoked are of a very poor quality.

The use of a tooth brush is by no means common amongst children. Even when they do brush their teeth, they are apt to do so at very irregular intervals. Again, many of the dentifrices in use are harmful if applied too frequently.

These are some of the more important causes of bad teeth in young persons of factory age. They are all remediable, as we shall point out later on, by very simple means.

In adult workers the condition of the teeth is, so far as my experience goes, even worse than in the juveniles. In other words, the harvest of neglect in youth begins to be reaped when manhood and womanhood are attained. During the war I examined a number of women who were seeking government appointments. These women were drawn from all classes of the community. A large proportion belonged to the domestic servant group. Amongst these it was rare to meet with really good teeth. Many wore artificial sets, while a minority had had the carious teeth extracted before examination. In the majority of instances, however, the teeth were defective and some had even to be rejected on that account. It is well known that domestic servants are specially liable to suffer from indigestion and other stomach troubles. It is little wonder, when we bear in mind how many of this class have defective teeth. But domestic servants are only one group of female workers. The same thing I found to be the case in factory girls, tailors' machinists, and perhaps to a less extent in typists and clerks. All female workers examined displayed, as a whole, a very low standard as regards the condition of the teeth.

In male workers conditions are extraordinarily bad. Out of 200 men recently examined, I did not find ten who had what one could term satisfactory teeth. Pyorrhea alveolaris was extremely common. In many cases there was recession of the gums, and tartar-covered teeth. Several workers had only a few stumps left, and these were carious. It is a remarkable fact, whether related to the condition of the teeth or not I am not prepared to say, that many of these 200 male workers had thickened arteries. Certainly the condition of the teeth is striking and instructive. It is hardly conceivable that I had discovered a special group of men. Rather, I think, we may take it that all male workers have more or less defective teeth.

If my contention that the teeth of the worker, male and female, juvenile and adult, are today in a very bad condition, then surely it is our duty to do something to rectify this state of matters in view of the facts that bad teeth have a most disastrous influence on the health of the worker and that they may even produce disease which may interfere with his work. Bad teeth may, therefore, prove to be a cause of lessened production. Accordingly, both from the worker's and the employer's point of view, as well as in the national interests, it is our duty to attend to the teeth of the worker.

What steps are to be taken in order to improve the condition of the teeth in the worker? At the very outset, prevention is best; prophylactic measures must be taken. In fact, much of the machinery for this exists, but it needs oiling. The school authority in every district should see to it that no boy or girl leaves school without having had the teeth attended to by a qualified dental surgeon. So far as my experience goes, parents are often to blame for not carrying out the suggestion of the school medical officer as regards their children's teeth. It is their bounden duty, however, to give every heed to this matter, and no stone should be left unturned to demand that such be the case. It is often too late when the young person enters a factory to have the teeth seen to. They may be beyond the reach of mere preventive measures then, and only extraction is possible. My contention is, therefore, that if a child has his teeth or her teeth attended to during school life there will be, in due time, a remarkable improvement in the condition of the teeth of the worker. This, therefore, is the first step in the ladder of progress towards the goal at which we aim.

When we find a boy or girl of factory age with defective teeth, what is to be done? At present all we can do is to recommend the young person to tell his parents that the teeth should receive attention. This, I fear, is a measure of practically no value whatever; even if the parents are told, they will turn a deaf ear to the advice thus proffered them gratuitously. What I think is wanted is legislation on the subject. It should be made compulsory by law that no boy or girl who is considered by the factory surgeon to have defective teeth should be allowed to commence work until such time as a certificate has been granted by a dental surgeon that the teeth have been thoroughly treated. The importance of looking after their children's teeth would thus be impressed on parents, who would probably, from this cause alone, cease to neglect the advice of the school medical officer. In this way the law made to regulate the condition of the factory worker's teeth would very soon cease to be actually necessary, although it would still remain as a safeguard against neglect.

In some of the larger factories, especially in those which employ a welfare worker, the teeth of every young person are attended to as soon as the worker is engaged. In some cases this is done at the instigation of the factory surgeon, but in many instances the young person is taken by the welfare supervisor to a dental surgeon for his advice. In our opinion, every factory employing 1,000 workers or more should have a medical man on its staff whose advice is available in case of need. Naturally, the local factory surgeon, who is fully acquainted with factory conditions, is the most suitable man for such work. Such factories should also have a dental surgeon, who would co-operate with the factory surgeon, and who should be paid either a fixed annual salary or receive so much on account of each worker employed. Smaller factories might combine for this purpose, and share a medical man and dental surgeon between them.

Cigarette smoking amongst boys should be prohibited by law, and fines ought to be inflicted on boys who continue to disregard the injunction. No boy should be allowed to smoke before the age of 18. I am aware that there are certain restrictions regarding smoking amongst children of school age, but this is evidentely a dead letter, as I have seen boys coming out of school lighting cigarettes under the eye of a passing policeman! What we want, and must insist on having, is a law which shall be obeyed and provisions of which will be carried out by those concerned.

Cigarette smoking is not only bad for the teeth, but it checks growth and retards gain in weight. I find that boys who smoke are pale, undersized, suffer from giddiness and loss of appetite, and usually have carious teeth.

So far as adults are concerned, a system of regualr inspection of workers in factories is necessary. In this article I am only dealing with the teeth of the worker, but eyesight, hearing and conditions of the circulatory and respiratory systems also demand attention. I maintain that every large factory should have a system of regular medical inspection of the employes, and smaller factories could easily combine for this purpose. It must often happen that workers suffering from curable diseases go on working until it is too late, quite oblivious to the fact that they are unfit. Much might be done, therefore, by securing the services of a medical man acquainted with factory life and the conditions of factory labor. One of his first duties would be to advise the worker to attend to his teeth, if they are found to be defective. It is one thing, however, to advise: it is quite another to get the worker to carry out your advice. If, however, there existed suitable machinery at the factory for attending to the teeth, much of the difficulty would be removed. At large works, therefore, a dental surgeon should attend as often as may be necessary in order to treat the dental defects of the workers, who would soon become reconciled to the new order of things when one or two of their companions had submitted themselves to the ordeal.

Much of the money that has been and will be wasted in administering the National Health Insurance Act might be diverted to subsidizing the medical and dental supervision of factory workers. This is a preventive measure which should appeal to that newly-constituted body, the Ministry of Health. We can only expect good work when we have really good workers. We can only have good workers when they are healthy and are *kept* in a healthy condition. To secure this, one of the first measures, we take it, is to attend to the teeth of the worker. This is a national duty which the state, that is, the income taxpayer, should see to. By curtailing unnecessary expenses in the various departments connected with the nation's health, and by thus liberating money enough to pay for medical and dental supervision of factory workers, great benefit would result. It is sad to think that so much money has been squandered during the war, money which would have sufficed to provide the whole country for years to come with well-paid medical men and dental surgeons. As it is, we have the government contracting for a few shillings a year, in return for which medical men in its service are called upon to treat the worker. All this must be changed. The Ministry of Health will not bring about such reforms as we have suggested. The employer, however, has it in his power. He is the man who finances the state, and it is to him that the state must look for its very existence. A strong representation made by the employers of labor throughout the country could bring about the needed reforms in a very short time.

The health of the worker in the factory must be attended to. So far little has been done in this direction. The appointment of welfare supervisors is a step forward, but it stops short of what we must aim at. The workers themselves usually welcome reforms when these are found to be of practical and personal benefit. I am satisfied that it would not take long for them to discover that measures taken to improve the condition of their teeth were helpful in giving them better health. The worker today is not so shortsighted as to overlook the advantages of reforms made in his interests. Such reforms as we have suggested are badly needed at the present time, and in bringing them before the notice of employers and others interested we feel that we are opening a door which hitherto has remained closed.

